# Comparison of surface wind speed and wind speed profiles in the Taklimakan Desert

**DOI:** 10.7717/peerj.13001

**Published:** 2022-04-01

**Authors:** Xinchun Liu, Yongde Kang, Hongna Chen, Hui Lu

**Affiliations:** 1 Institute of Desert Meteorology, China Meteorological Administration, Urumqi, China; 2 Taklimakan National Field Scientific Observation and Research Station of Desert Meteorology, Tazhong, China; 3 Xinjiang Key Laboratory of Desert Meteorology and Sandstorm/ Key Laboratory of Tree-ring Physical and Chemical Research, China Meteorological Administration, Urumqi, China; 4 Xi’an University of Technology, Xi’an, Shanxi, China; 5 Department, Urumqi Environmental Monitoring Center, Urumqi, Xinjiang, China; 6 Guang Xi Normal University, Nanning, Guangxi, China

**Keywords:** Desert hinterland, Surface layer, Diurnal variation, Profile, Taklimakan Desert

## Abstract

Near-surface (10 m) wind speed (NWS) plays a crucial role in many areas, including the hydrological cycle, wind energy production, and the dispersion of air pollution. Based on wind speed data from Tazhong and the northern margins of the Taklimakan Desert in Xiaotang in spring, summer, autumn, and winter of 2014 and 2015, statistical methods were applied to determine the characteristics of the diurnal changes in wind speed near the ground and the differences in the wind speed profiles between the two sites. The average wind speed on a sunny day increased slowly with height during the day and rapidly at night. At heights below 4 m the wind speed during the day was higher than at night, whereas at 10 m the wind speed was lower during the day than at night. The semi-empirical theory and Monin–Obukhov (M–O) similarity theory were used to fit the NWS profile in the hinterland of the Tazhong Desert. A logarithmic law was applied to the neutral stratification wind speed profile, and an exponential fitting correlation was used for non-neutral stratification. The more unstable the stratification, the smaller the *n*. Using M–O similarity theory, the “linear to tens of” law was applied to the near-neutral stratification. According to the measured data, the distribution of *φ*_*M*_ with stability was obtained. The *γ_m_* was obtained when the near-surface stratum was stable in the hinterland of Tazhong Desert and the *β_m_* was obtained when it was unstable. In summer, *γ*_*m*_ and *β_m_* were 5.84 and 15.1, respectively, while in winter, *γ_m_* and *β_m_* were 1.9 and 27.1, respectively.

## Introduction

Near-surface (10 m) wind speed (NWS) and temperature are important parameters for studying atmospheric dynamics and climate change. Research on wind speed and temperature changes will improve our understanding of atmospheric circulation, leading to better climate analyses and predictions. The NWS is one of the key variables in climate research. Changes in the NWS have significant implications for human society and the natural environment (Pryor, Schoof & Barthelmie, 2006). The intensification of NWS may aggravate soil erosion, resulting in more severe sandstorms (Alizadeh-Choobari, Zawar-Reza & Sturman, 2014; Wang et al., 2017). Over the past decade, scientists have conducted studies to determine the trends in wind speed at the height of 10 m near the surface (McVicar et al., 2012). Globally, wind speeds have recovered slightly since the 2010s, particularly in Central and East Asia (Kim & Paik, 2015; Dunn et al., 2016; Azorin-Molina et al., 2017; Ming, Wei & Wang, 2019). The observed upward trend in the global ocean surface has revealed the complexity of decades of wind speed changes (Wentz et al., 2007; Tokinaga & Xie, 2011; Young, Zieger & Babanin, 2011). In addition, variations in wind speed above and below the boundary layer suggest many uncertainties behind the stationary phenomenon. As a product of the atmospheric boundary layer, near-surface wind is transient in nature and is affected by topography and boundary layer processes (Mahrt, 2009; Belušić & Güttler, 2010; Güttler & Belušić, 2012).

Studies of the atmospheric boundary layer in arid regions are an important part of climate research in arid regions (Li et al., 2017). Similarity theory is one of the most important analysis and research methods in atmospheric boundary layer meteorology. After establishing the relatively mature Monin–Obukhov (M–O) similarity theory for describing the atmospheric near-surface layer, researchers have attempted to develop a similar similarity theory for the whole boundary layer. The atmospheric boundary layer is also the main characteristic quantity for assessing turbulent mixing, vertical disturbance, convective transmission, cloud belts, atmospheric pollutant diffusion, and analyzing atmospheric environmental capacity (Therry & Lacarrere, 1983; Holtslag & Boville, 1993; Hong & Pan, 1996; Beyrich, 1997; Collier et al., 2005). Earlier studies of the boundary layer mainly focused on the near-surface layer. The development of turbulence theory and technological developments in measuring atmospheric processes have promoted research on the atmospheric boundary layer (Monin & Obukhov, 1954; Johnson, Williams & Shepherd, 2021). The structure of the atmospheric boundary layer and its evolution have significant diurnal characteristics. After sunrise, solar radiation heats the ground. The increase in the heat flux generated by the near-surface layer strengthens turbulent mixing, the height of the atmospheric boundary layer increases, and the heat content of the boundary layer also increases. It mixes uniformly and the wind speed, temperature, and specific humidity change little with height. This is referred to as the mixed layer. After sunset, long-wave radiation is emitted from the ground, which cools down, resulting in a weakening of turbulent transport. The near-surface layer forms a stable boundary layer, and the upper mixed layer rises from the ground. During this uplift, the atmospheric turbulence characteristics are significantly weakened, but the distribution of meteorological elements in the mixed layer during the day are still maintained. This layer is obviously different from the upper free atmosphere, and is called the residual layer. Because there is often an inversion layer on the top of the atmospheric boundary layer, the upward development of turbulent mixing is inhibited, and therefore a boundary is formed between the atmospheric boundary layer and the free atmosphere. Due to the differences in the thermal properties of the topography and underlying surface, the height of the atmospheric boundary layer presents obvious spatial variation characteristics. Although the Taklimakan Desert in China and the Pearl River Delta have similar frequencies of boundary layer occurrence, the average atmospheric boundary layer height in the Taklimakan Desert is significantly higher (Zhang et al., 2017). Therefore, in different locations and under different weather backgrounds, the height of the boundary layer can be very different.

With the completion of the ecological protection barrier along the highway of the Taklimakan Desert and the restored green spaces of the oil base in the hinterland of the Taklimakan Desert, the nature of the regional underlying surface has changed, resulting in changes in surface wind speed. In the context of climate change, the Taklimakan Desert climate is essentially a complex of basin and desert climates, resulting in an extreme arid continental climate.

This study used wind speed data collected by the gradient system of the Taklimakan Desert Tazhong and Xiaotang 10 m meteorological observation towers to analyze the characteristics of the wind speed diurnal variation in the surface layer at different observation stations in the Taklimakan Desert from August 2014 to May 2015. A comparison of the exchange rates between different wind speed profile models in the Taklimakan Desert provided observational data for the development of near-surface atmospheric observation experiments and atmospheric environment research in the Taklimakan Desert. This will be useful for understanding the factors controlling the microclimate of the farmland around the Tarim Basin, improving the near-surface model of the soil-vegetation-atmosphere system, and better understanding the local agrometeorology. The level of technology in the area was of great significance in the study. The special application (*e.g*., climatological evaluations for a large catchment area, event-based short-term analyzes, improving special models etc.) significantly influences the selection of the necessary data base and analyzing methods. Tarim Oilfield is in the desert hinterland. With the development of oil and gas resources, the study of desert boundary layer climate is particularly important. Therefore, the research conclusions of this paper can provide a certain scientific reference for climate change and economic development of cities around deserts, and can also better serve oil, natural gas and other industries.

## Materials and Methods

**Figure 1:**
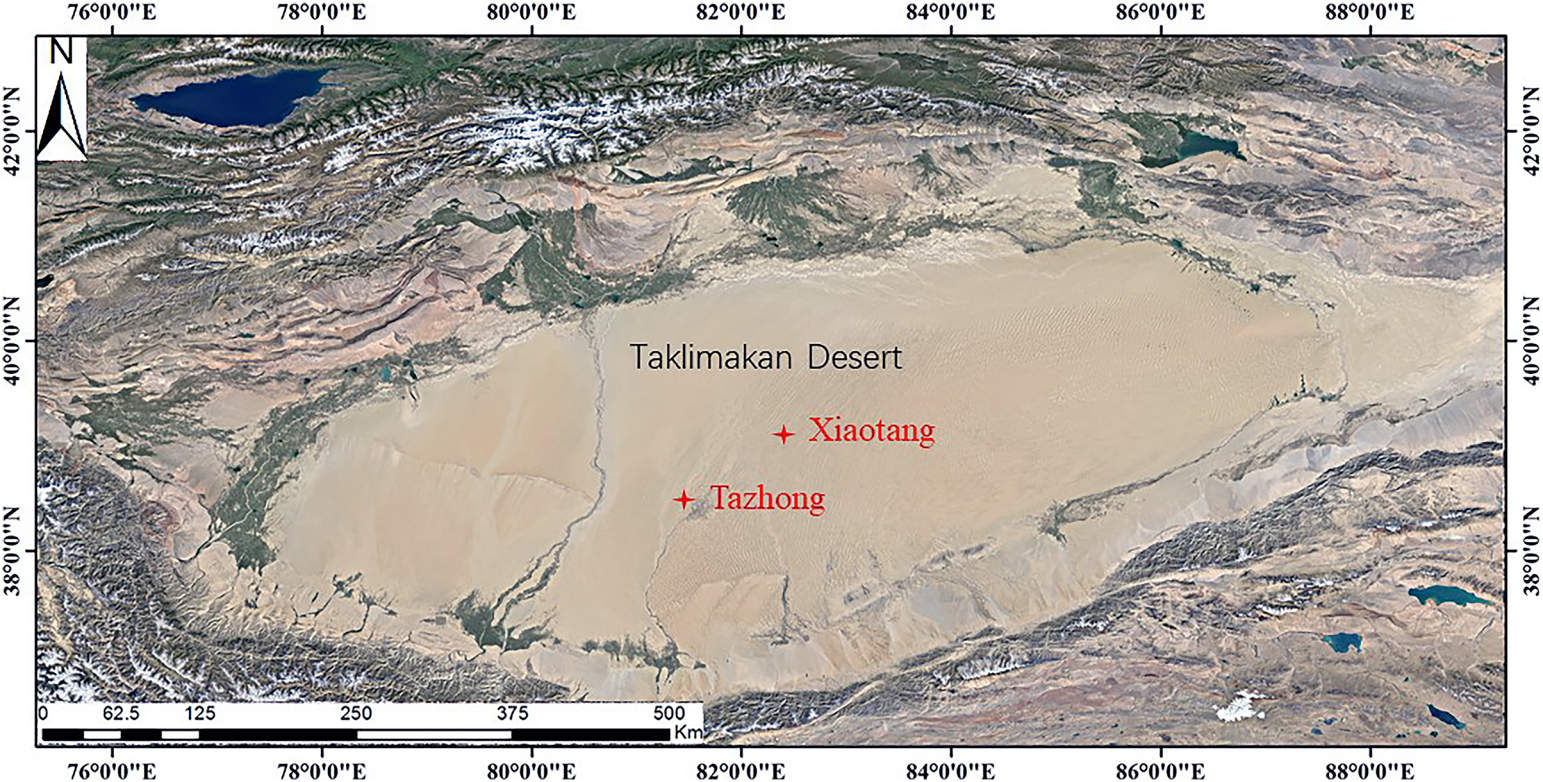
Map of the study area.

The Taklimakan Desert, located between the Tianshan and Kunlun Mountains, is a closed inland basin. While the east of the desert is bounded by the Gobi Desert, the other three sides are surrounded by mountains, including the southern Tibetan Plateau, the Pamir Plateau in the southwest, and the Tianshan Mountains in the north and northwest. Due to this special terrain and geographic location, it is difficult for ocean currents to penetrate the Tarim Basin. The Taklimakan Desert therefore has a typical continental climate, with an abundance of light and heat, little precipitation, strong sunshine, sandy weather, and large temperature differences between day and night, and the different seasons. In this study, two typical observation stations were selected in Xiaotang and Tazhong (Fig. 1). Xiaotang observatory (40°48.126′N, 84°18.211′E, altitude 912 m) is located on the northern edge of the Taklimakan Desert, about 40 km from the Tarim River. It is a typical desert hinterland-desert-oasis transition zone. The underlying surface is flat sandy land, with ancient river bed in some areas, and there is no vegetation. The station is located on the south bank of an ancient river bed. An area of mobile dunes is located about 250 m to the south, and mainly consists of crescent dunes and compound crescent dune chains. The dune landform belongs to the ancient Tarim River alluvial-flood plain, and is the intersection of two desert areas. The area has an inland warm temperate desert climate, which is subject to drought and little rain. The annual average wind speed is 2.5 m·s^–1^, with a maximum in spring and summer, and a minimum in winter. The change of air temperature at Xiaotang station is similar to that of wind speed, with the maximum in June–July and the minimum in December–January. The change of wind speed and air temperature has an obvious synchronization. Most (80%) sandstorms occur in spring and summer, with the lowest frequency in winter. The annual average wind speed is 2.8 m/s, and the average temperature is 11.2 °C. Tazhong is located in the center of the hinterland of the Taklimakan Desert (83°39′E, 38°58′N), with the highest temperature in summer of 46.0 °C, and the lowest temperature in winter of –25.0 °C. The average temperature of the four seasons is 13.6 °C, the average annual precipitation is about 25.9 mm, and the evaporation is about 3,812.3 mm. The regional climate is abnormal, the vegetation coverage is extremely low, and only cold and drought-resistant shrubs (*e.g*., *Haloxylon ammodendron*) can survive. The peak period of dust storms is from March to August every year. The frequency of dust storms at 12 m above the ground is about 500 per year. The normal average wind speed is 2.5 m·s^–1^, and the sand activity intensity index reaches a maximum value of about 8,000 each year. The prevailing wind direction is northeast and northwest.

**Figure 2:**
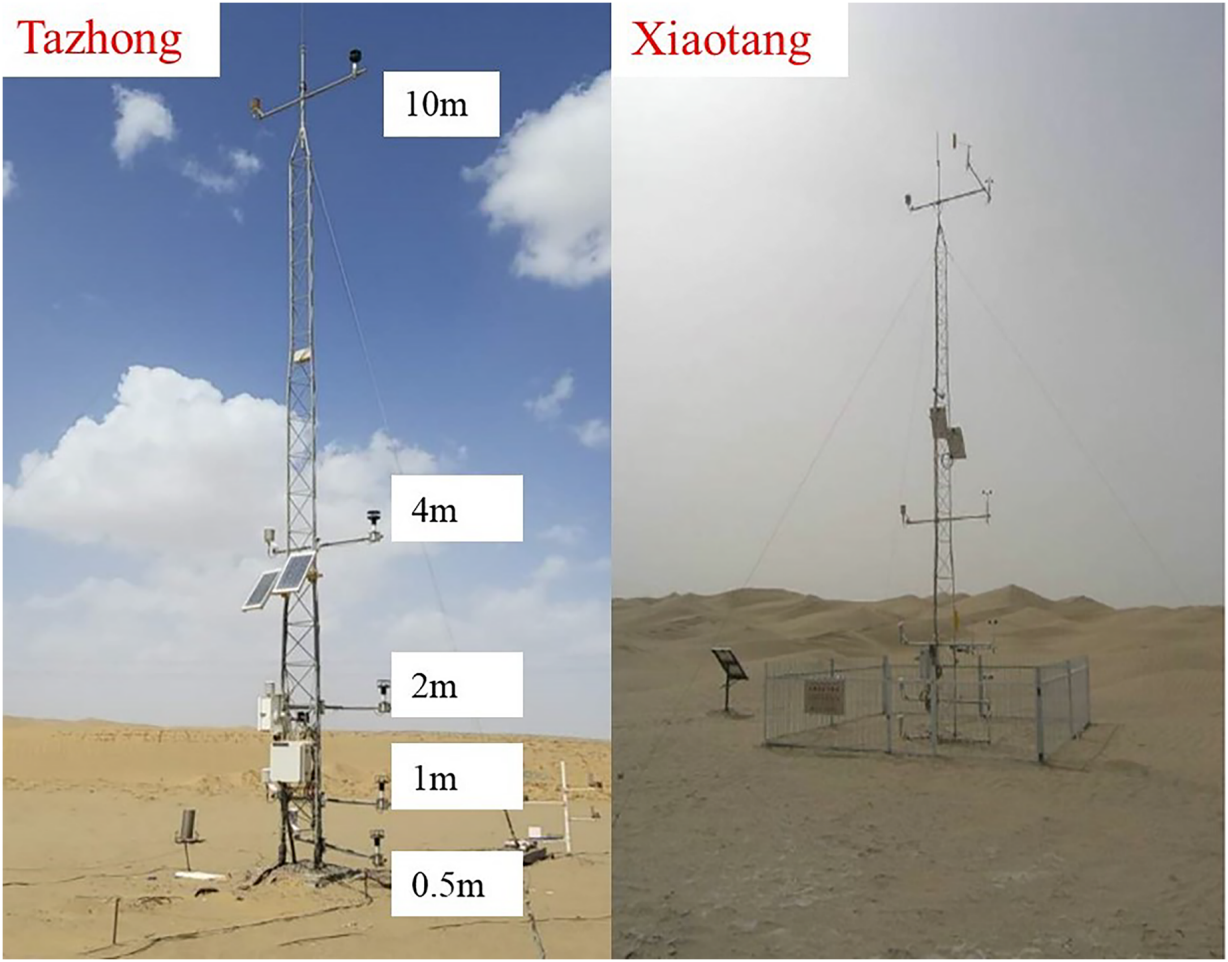
The 10 m observation meteorological towers in Tazhong and Xiaotang.

In this study, a windsonic two-dimensional ultrasonic wind speed and direction sensor (1590-PK-020, Campbell Scientific, Logan, UT, USA) and temperature and humidity sensor (1590-PK-020, Campbell Scientific, Logan, UT, USA) installed on meteorological observation towers were used to conduct parallel comparative experimental observations of microclimate elements in Tazhong and Xiaotang from January 1, 2014 to December 31, 2015 (Fig. 2). The wind speed and direction sensor had a starting wind speed of 0.01 m/s; precision of wind speed ±2%; range of 0–60 m/s and 0–359°; and resolution of 0.01 m/s and 1°. The range of the temperature sensor was –80–60 °C, the accuracy was ±0.17 °C, and the resolution was 0.1 °C. The wind speed, temperature, and relative humidity data used in the study were obtained at the height of 10 m. The data used were subjected to quality control, including the synchronous calibration of the two observation points, the logical extremum of observation data, and a time consistency check. In this study, the four seasons were classed as spring (March to May), summer (June to August), autumn (September to November), and winter (December to February). January, April, July, and October were the representative months of winter, spring, summer, and autumn, respectively.

## Results

### Diurnal variations of NWS

**Figure 3:**
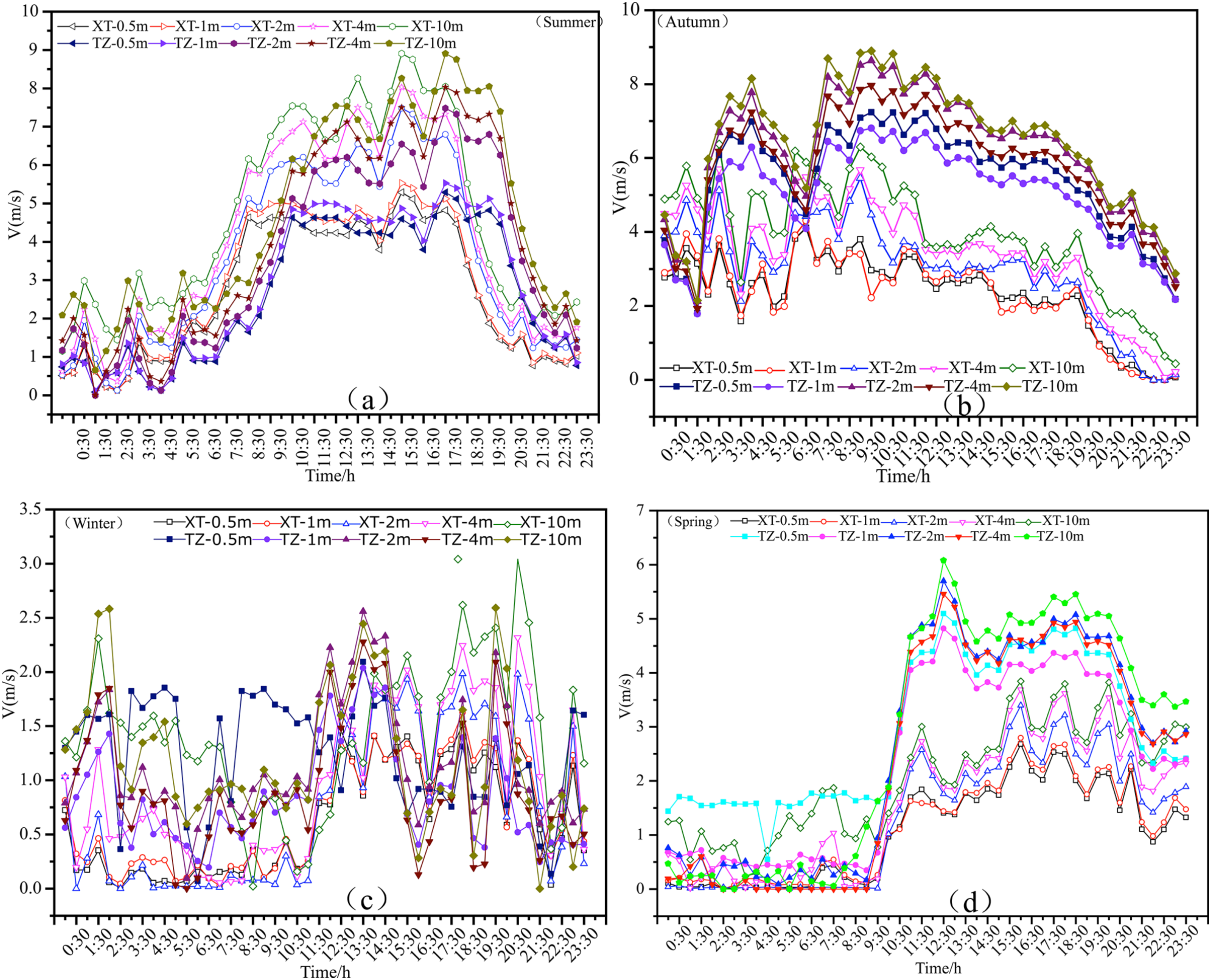
Diurnal variations of NWS in Tazhong and Xiaotang.

Figures 3(A)–3(D) show the diurnal variations of mean wind speed in summer, autumn, winter, and spring. Using the ground meteorological data, typical sunny days in each seasonally representative month were selected to determine an average wind speed per hour. The sunny days in summer were August 27, 28, and 31, 2014, the sunny days in autumn were October 3, 10, and 11, 2014, the sunny days in winter were January 4, 6, and 7, 2015, and the sunny days in spring were April 12, 13, and 15, 2015. Based on the average daily variation of the surface layer height in the four seasons, it was found that the daily variation of wind speed in Tazhong and Xiaotang on sunny days had two characteristics. First, there were two peaks and two low values in the daily variation of wind speed. The two peaks occurred at night and in the daytime, and the two low values occurred in the morning and evening, respectively. However, the magnitude and occurrence of wind speed were different in the different seasons. Second, there were two distinct forms of change in the lower and upper layers. In the lower layer, the daytime wind speed was greater than in the night, in the upper layer the nighttime wind speed was greater than during the daytime, and the middle layer was stable, but there were differences in each season.

#### Diurnal variations of NWS in summer

The diurnal variations of surface wind speed in Tazhong and Xiaotang in summer are shown in Fig. 3(A). The wind speed at different altitudes from 0.5 to 10 m displayed an increasing trend. The maximum wind speeds in each layer in Tazhong and Xiaotang were similar, with values of 5.286, 5.525, 7.479, 8.030, and 8.905 m/s at 0.5, 1, 2, 4, and 10 m, respectively. From 0.5 to 2 m, the highest wind speed occurred at 3:00 at night and 15:30 in the daytime, and the lowest wind speed occurred at 0:30 and 23:30 at night. At 4 and 10 m, the wind speed in Xiaotang was larger than that in Tazhong at 0:30–13:30, and the wind speed difference was about 1–2 m/s. However, the situation at 13:30–23:30 was the complete opposite, with the wind speed in Tazhong being greater than that in Xiaotang. At 0.5–2 m, the situation between the two sites at 17:30–23:30 was also the opposite. Comparing the diurnal variation curves of wind speed at different altitudes, it was found that the wind speed increased continuously from 0.5 to 1 m. The wind speed at 0.5 m was below 5 m/s, while the wind speed at 2 m was always above 4 m/s.

After sunrise (07.00) in the morning, due to the increasing solar altitude angle and the strengthening of turbulence exchange, the wind speed in each height layer increased rapidly. The average increase of wind speed in each height layer from 0.5 to 10 m was about 0.9 m/s per hour, until 10.00. However, the wind speed at Xiaotang and Tazhong in the 0.5 and 1 m layers displayed a sharp downward trend and then increased slightly until 16:30. At 2, 4, and 10 m, the wind speed increased relatively quickly until 19:00, at which point the wind speed began to decline sharply, leading to a diurnal difference in wind speed of about 6 m/s. After 18.00, with the decreasing solar altitude angle, the ground radiation balance decreased rapidly, the turbulence weakened, and the wind speed decreased rapidly. The decreasing trend in NWS was most obvious below 4 m, with the average wind speed decreasing by 1 m/s per hour. Due to the gradual formation of a near-surface inversion, the turbulence was further weakened, and the wind speed below 10 m continued to decrease, reaching a minimum at 21.00, with an average wind speed of less than 1 m/s from 21.00 to 22.00. This may be because the valley between the large sand ridges alongside the tower was associated with downhill winds at this time.

#### Diurnal variations of NWS in autumn

Figure 3(B) shows the diurnal variations of wind speed at 10 m height in Xiaotang and Tazhong in autumn, with the results being similar to those in summer. Overall, the diurnal variation of wind speed in Tazhong was greater than that in Xiaotang, especially in the 2, 4, and 10 m height layers from 09:30 to 23:30. In both Xiaotang and Tazhong, at 12:30 in the autumn the wind speed was high in the daytime at low levels and low in the nighttime at high levels. Compared with summer, the average wind speed in autumn was slightly lower. At 2, 4, and 10 m, the highest wind speed occurred at 03:30 at night and 09:30 in the daytime, with values of 7.9 and 8.79 m/s, respectively. The lowest wind speed occurred at 06:00, with values of 4.8 and 2.3 m/s, respectively. The difference between the maximum and minimum daily wind speed at 10 m was 3.5 m/s. The time at which the maximum wind speed occurred in Xiaotang was the opposite of that in Tazhong, *i.e*., the time of the minimum wind speed in Tazhong was the time of maximum wind speed in Xiaotang. The highest wind speeds at 10 m in Xiaotang occurred at 01:00 and 08:30 pm in the daytime, with values of 5.81 and 6.9 m/s, respectively. The lowest wind speeds occurred at 03:00 and 23:30, with values of 2.1 and 1.8 m/s, respectively. The average daily maximum wind speed was only 2.8 m/s larger than the minimum wind speed, and the daily variation of the low-level wind speed was much reduced compared with that in the higher layer.

At 08:00, the NWS within 10 m in Tazhong and Xiaotang decreased rapidly, and at 15:30, the wind speed in each altitude layer decreased significantly. The decrease in Tazhong was greater than that in Xiaotang, with a difference of about 4 m/s, which was different from the pattern observed in the summer. In Tazhong, the wind speeds remained high but the wind speed curves at all five levels in the autumn daytime were more closely arranged than those in Xiaotang, indicating that the wind speed difference in the surface layer within 10 m during the autumn daytime was smaller than that in summer. The wind speed difference between the highest and lowest layers in autumn was 1–2.2 m/s, which was about 0.5 m/s smaller than that in summer.

#### Diurnal variations of NWS in winter

As shown in Fig. 3(C), the diurnal variation of wind speed in the 10 m layer of Tazhong and Xiaotang in winter was generally small, and the wind speed in the whole 10 m layer was less than 3 m/s. In the Xiaotang area, below 4 m from 00:30 to 09:00 the wind speed was maintained at about 0.2 m/s. At 09:30, the wind speed began to increase, until at 23:30 it reached about 1.5 m/s. The wind speed trends in Tazhong and Xiaotang followed the completely opposite trend. From 00:30 to 09:00 the wind speed at 0.5 m was greater than in the other layers, with a maximum of about 1.8 m/s. In winter, the average wind speed in the daytime was larger than that in the nighttime above 2 m. The highest wind speeds in Tazhong and Xiaotang generally occurred at 13:00 and 19:30 in the daytime, and the lowest wind speeds occurred at 21:00 and 06:00, respectively. The highest wind speeds at 10 m were 2.5 and 2.9 m/s, and the lowest wind speeds were 0.2 and 0.02 m/s, respectively. The average wind speeds at night in Tazhong and Xiaotang from 0.5 to 1 m were higher than in the daytime. The difference between the maximum and minimum wind speed at 10 m was only 1.3 m/s, which was smaller than in summer and autumn. After 10:30 in winter, the wind speed at each altitude increased slowly. This increase was small and occurred about 2 h later than in summer. After 10.00, the wind speed began to increase at 2 m, and then began to decrease until 16:30. This feature also occurred at 09:00 in autumn at 2 m.

#### Diurnal variations of NWS in spring

Figure 3(D) shows the diurnal variations of surface wind speed in Tazhong and Xiaotang in spring. Similarly, to summer, the wind speed was high in daytime and low at night within 10 m. The wind speed in each layer in Tazhong from 09:30 was obviously larger than in Xiaotang, with the maximum difference in each layer being about 1–3.3 m/s. From the morning of the previous evening to 09:30 the next day, the trends in wind speed changes in Tazhong and Xiaotang were similar, with a stable transition and wind speed difference of about 0.9 m/s. The difference between the maximum and minimum daily average wind speed at each height in the tower was 5.3–6.1 m/s. The difference between the maximum and minimum daily average wind speed in each layer in Xiaotang was 2–3.1 m/s. The diurnal variations of wind speed in the 0.5–2 and 2–4 m layers were similar. From 09:00 to 12:30 in the morning, with the increase in the solar altitude angle, the turbulence exchange was enhanced and the wind speed increased rapidly. In the next 2 h, the wind speed at 1 m increased from 0.5 to 4.1 m/s, at 2 m it increased from 0.3 to 4.5 m/s, at 4 m it increased from 0.1 to 5.8 m/s, and at 10 m it increased from 0.5 to 6.1 m/s. After 18:30, the wind speed at all altitudes showed a downward trend, although this was less obvious than that in summer and autumn. From midnight to sunrise, the NWS within 10 m was much smaller than that in summer, and was similar to that in winter.

### Comparison of the different wind speed profiles

A curve fitting of the variation in wind speed with height was applied to enable an understanding the near-surface meteorological characteristics in the hinterland of the Taklimakan Desert, as well as an in-depth understanding of the desert wind-sand movement law, and the development and utilization of wind energy resources. It is important to note that in the subsurface the wind direction does not change with height, and therefore the effect of Coriolis force in the subsurface can be ignored. The most commonly used methods for fitting the wind speed profile are the logarithmic law and the exponential law model. Based on a semi-empirical theory, an improved model of the variation of wind speed with height was established according to the M–O similarity theory.

#### Wind speed profile model under a semi-empirical theory

##### Wind speed profile under neutral stratification

Thermal turbulence does not develop in neutral stratified air, with turbulent motion being completely dependent on the role of dynamic factors. Turbulent motion in the near-surface layer is very similar to the simulated turbulence in a wind tunnel. The relationship $l = kz$ between the mixing length $l$ and the vertical height $z$ from the ground in a wind tunnel experiment was introduced into a near-surface layer under neutral stratification, where ${k}$ is the Karman constant (generally 0.4).

The relationship between shear stress and mixing length, and the average wind speed was substituted into $l = kz$:

(1) $$\tau=\rho {\kappa ^2}{{\rm z}^2}{\left( {\displaystyle{{\partial \overline { u} } \over {\partial z}}} \right)^2}$$

By introducing the friction velocity ${{ u}_*}$, it was considered that ${{ u}_*} = \sqrt {{\tau \over \rho }}$ did not change with height in the near-surface layer, and Eq. (1) was rewritten to Eq. (2):

(2) $$\displaystyle{{\partial \overline { u} } \over {\partial z}} = \displaystyle{{{u_*}} \over {kz}}$$

The pairwise Eq. (2) integral is:

(3) $$\overline { u} = \displaystyle{{{u_*}} \over k}\ln z + A$$

$A$ is an integral constant. Many experiments have found that the height at which the average wind speed is zero typically occurs at a height *z* from the ground that is referred to as ${{z}_o}$.

If boundary conditions are applied: *z* = *z*_0_, $\overline u = 0,$ Eq. (3) can be written as:

(4) $$\overline { u} = \displaystyle{{{u_*}} \over k}\ln \displaystyle{z \over {{z_0}}}$$

Equation (4) expresses a typical NWS profile under neutral stratification as a logarithmic wind speed profile.

**Figure 4:**
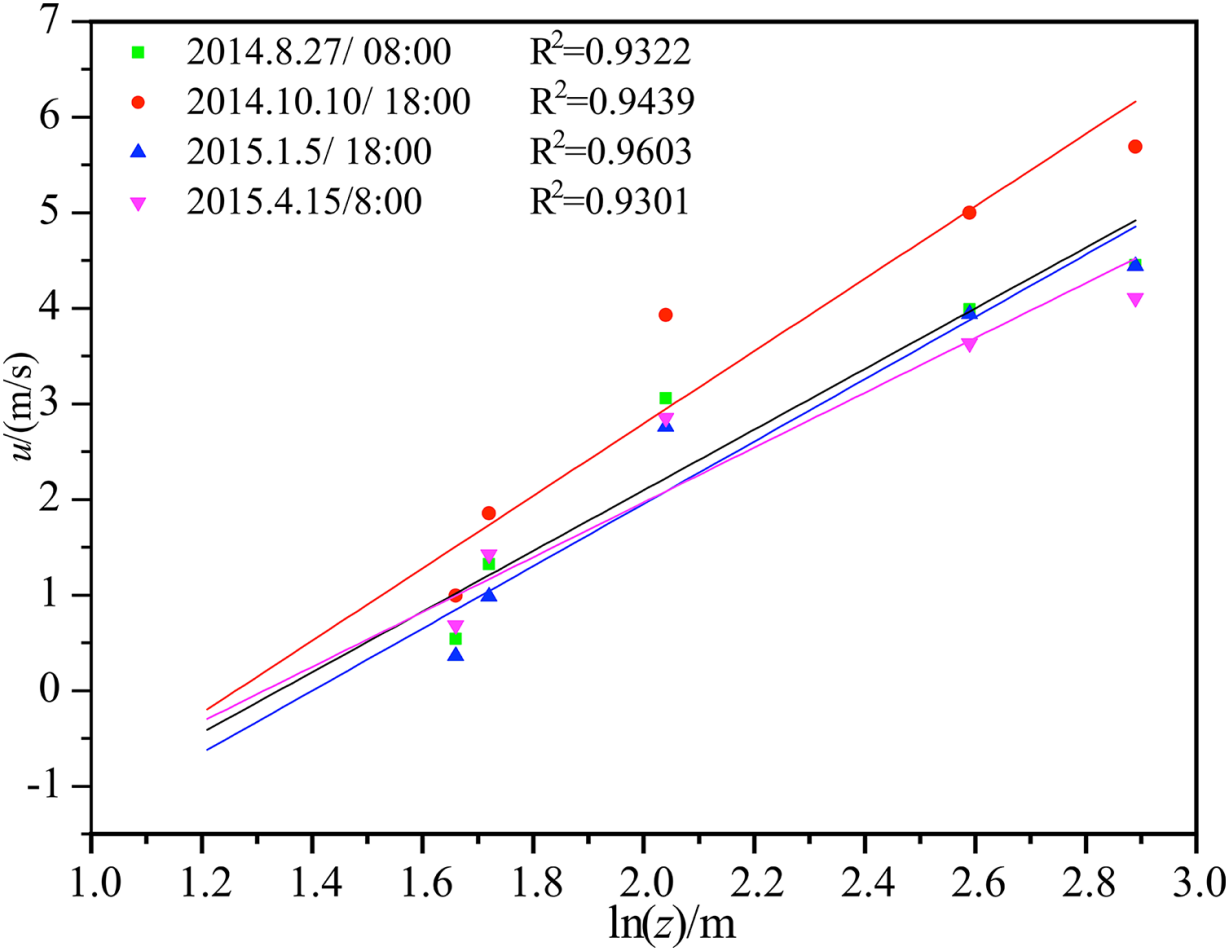
Average wind speed and logarithmic law curve fitting under neutral stratification in the middle of Tazhong tower.

Figure 4 shows the average wind speed and logarithmic fitting under neutral stratification in the middle of the Tazhong tower, taking the $y$ axis as $\ln z$. It can be seen from the diagram that the near strata in the tower are neutral layers and are very suitable for expressing a logarithmic distribution, with large correlation coefficients. The time period of the neutral stratification in the atmosphere was consistent with the transition period of the temperature profile.

##### Wind velocity profile under non-neutral stratification

Under non-neutral stratification, the wind speed profile will deviate from the logarithmic law, and can be expressed by a simple exponential law in the form of:

(5) $$\overline { u} = \overline {{u_1}} {\left( {\displaystyle{z \over {{z_1}}}} \right)^n}$$

where *u* is the average wind speed at height *z* and *n* is the stability parameter, which is expressed as a positive fraction, 0 < *n* < 1.

**Figure 5:**
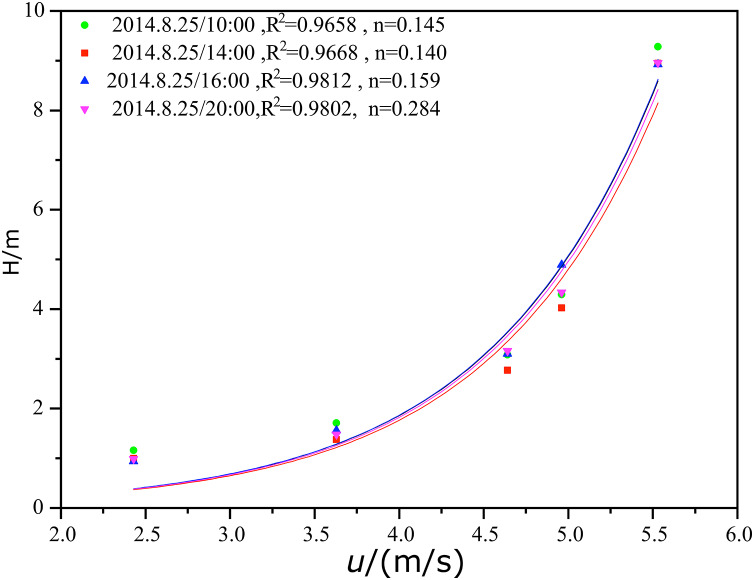
Fitting of the mean wind speed and exponential law under non-neutral stratification in the middle of Tazhong tower.

Figure 5 shows the fitting of the wind speed profile under non-neutral stratification in Tazhong on August 25, 2014 with the exponential law. The fitting results were very good, and the correlation coefficients were all above 0.98. The stability parameters of each period were fitted. The stability parameter *n* was smaller in the daytime than in the night, changing from 0.145 to 0.140 from 10:00 to 14:00 pm, indicating that the more unstable the stratification was, the smaller the *n* value was. After 20:00, the atmosphere gradually became stable with an *n* value of 0.284. With the gradual formation of a nighttime inversion layer, the *n* value increased, tending to 1.

#### The wind speed profile model established by M–O similarity theory

Under uniform and steady conditions, in the atmospheric layer where the wind direction was not significantly deflected with height, according to M–O similarity theory, the dimensionless wind speed gradient in the near-surface layer can be expressed as:

(6) $$\displaystyle{{kz} \over {{u_*}}}\displaystyle{{\partial u} \over {\partial z}} = {\varphi _M}\left( {\displaystyle{Z \over L}} \right)$$

where *k* is the Kaman constant (generally 0.4), ${{ u}_*}$ is the local friction velocity, and ${\varphi_M}$ is a general function. Under neutral stratification, ${\varphi_M} = 1$. According to the Businger–Nyer relationship, under non-neutral stratification the following expression applies:

(7) $$\eqalign{& {\varphi_M}\left( {\displaystyle{Z \over L}} \right) = 1 + {\beta _m}\displaystyle{Z \over L},\displaystyle{Z \over L}\rangle 0 \cr & }$$

(8) $$\eqalign{ {\varphi_M}\left( {\displaystyle{Z \over L}} \right) = {\left(1 - {\gamma _m}\displaystyle{Z \over L}\right)^{{1 \over 4}}},\displaystyle{Z \over L} \le 0 \cr  }$$

where *L* is the Obukhov length, and ${\beta _{\rm m}}$ and ${\gamma _{\rm m}}$ are empirical constants determined by measured values. Due to the different data sources, the values of the empirical constants are different.

##### The wind speed profile under near-neutral stratification

When the atmosphere is close to neutral stratification, $\left| {{Z \over L}} \right| \ll 1$. At this time, the general function is ${\varphi_M}\left( {{Z \over L}} \right) \to 1$. At the point where 0 is reached the formula is expanded according to the Taylor series and then the second order trace is omitted:

(9) $${\varphi_M}\left( {\displaystyle{Z \over L}} \right) \cong 1 + \beta \displaystyle{Z \over L}$$

(10) $$\displaystyle{{\partial \overline { u} } \over {\partial z}} = \displaystyle{{{u_*}} \over {kz}}{\varphi_M}\left( {\displaystyle{Z \over L}} \right)$$

Equation (10) is a two-sided integral, with boundary conditions, *z* = *z*_0_ , $\overline u = 0$. Equation (11) is the expression of the wind speed profile under near-neutral stratification:

(11) $$\overline { u} = \displaystyle{{{{ u}_*}} \over k}\left[ {\ln \displaystyle{z \over {{z_0}}} + \beta \displaystyle{{\left( {z - {z_0}} \right)} \over L}} \right]$$

Equation (11) adds only one highly linear term to the logarithmic contour, and is therefore referred to as the logarithmic + linear contour. Under neutral stratification, because ${ L} \to \infty$, the logarithmic + linear profile can be simplified to a logarithmic profile.

**Figure 6:**
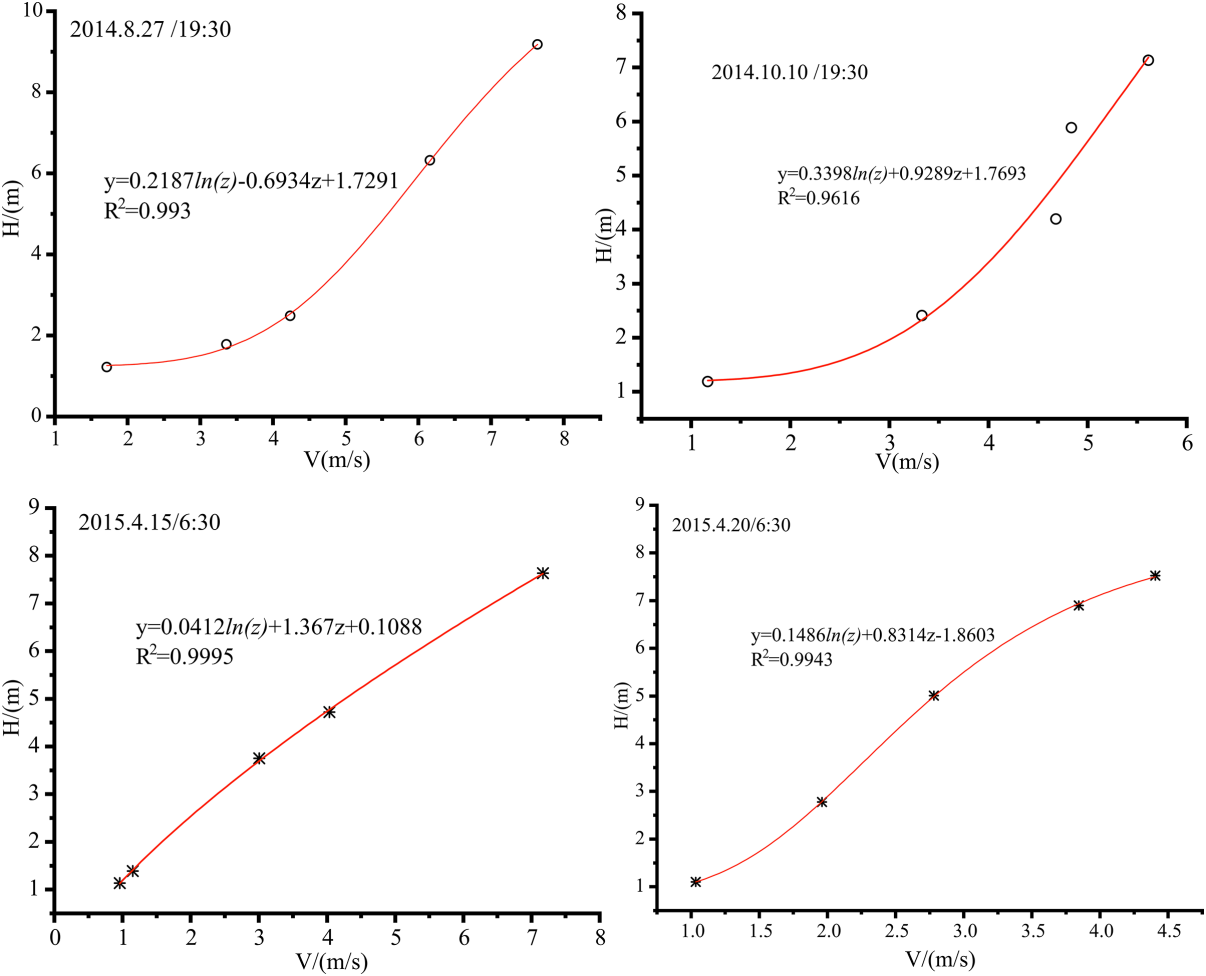
Pairwise linear fitting of the average wind speed under near-neutral stratification in the middle of Tazhong tower.

Figure 6 shows the wind speed profile fitted by the logarithmic + linear laws under near-neutral stratification in the Tazhong area. Generally, the atmospheric stratification was characterized by near-neutral conditions around sunrise and sunset, and the fitting results were also consistent with this period. The correlation coefficient from the fitting was large, indicating that the wind speed profile in a near-neutral atmosphere of weak stability and weak instability conditions in Tazhong satisfied the logarithmic dozens of linear laws.

##### The wind speed profile under stable stratification

In general, the wind profile theory under stable stratification is not satisfactory. The M–O similarity model is:

(12) $$\displaystyle{{kz} \over {{u_*}}}\displaystyle{{\partial u} \over {\partial z}} = 1 + {\gamma _m}\displaystyle{Z \over L}$$

Although stable stratification inhibits turbulence and the use of the M–O similarity model is limited, many NWS gradient observations have confirmed the reliability of the M–O model. For the low-level atmosphere above the constant flux layer, Yokoyama et al. verified that their generalized M–O similarity theory could be applied to the surface layer, and proposed a first-order approximate expression of ${u_*}$ that varied with height:

(13) $${u_*} = {u_0}{\left(1 - \displaystyle{z \over h}\right)^n} = {u_{o*}}{\zeta ^n}$$

where $h$ is the atmospheric boundary layer thickness and ${u_{0*}}$ is the friction velocity at the ground surface $\zeta = 1-\displaystyle{{\rm z} \over h}$.

According to Yamamoto (1979), in the layer where the wind direction has no obvious monotonic deflection with height, if the influence of Coriolis force is ignored, only the change of *u* with height should be considered. The differential equation obtained by Eqs. (12) and (13) is as follows:

(14) $$\displaystyle{{{ kz}} \over {{u_*}}}\displaystyle{{\partial u} \over {\partial z}} = (1 + {\gamma _m}\displaystyle{z \over {}}L){\left(1 - \displaystyle{z \over h}\right)^n}$$

##### The wind speed profile under stable stratification

Under unstable conditions, the concept of M–O similarity can be extended. Therefore, in this study *u* was still regarded as a constant from ${{ z}_0}$ to the $z$ integral,

(15) $${ u} = \displaystyle{{{u_*}} \over k}\left[ {\ln \displaystyle{z \over {{z_0}}} - {\varphi_m}\left( {\displaystyle{z \over L}} \right) + {\varphi_m}\left( {\displaystyle{{{z_o}} \over L}} \right)} \right]$$

In which, ${\varphi_{\rm m}} = \ln \left[ {{{1 + {x^2}} \over 2}{{\left({{1 + x} \over 2}\right)}^2}} \right] - 2\!\arctan x + {\pi \over 2}$, ${ x} = {\left( {1 - {\beta _m}{z \over L}} \right)^{0.25}} = \varphi_m^{ - 1}$.

The ${\varphi_m}$ value for the measured data at the tower height of 10 m was calculated using Eq. (6), and then the empirical constants ${\gamma _m}$ and ${\beta _m}$ in the tower were fitted according to the relationship between Eq. (7) ${\varphi_m}$ and ${z \over L}$.

**Figure 7:**
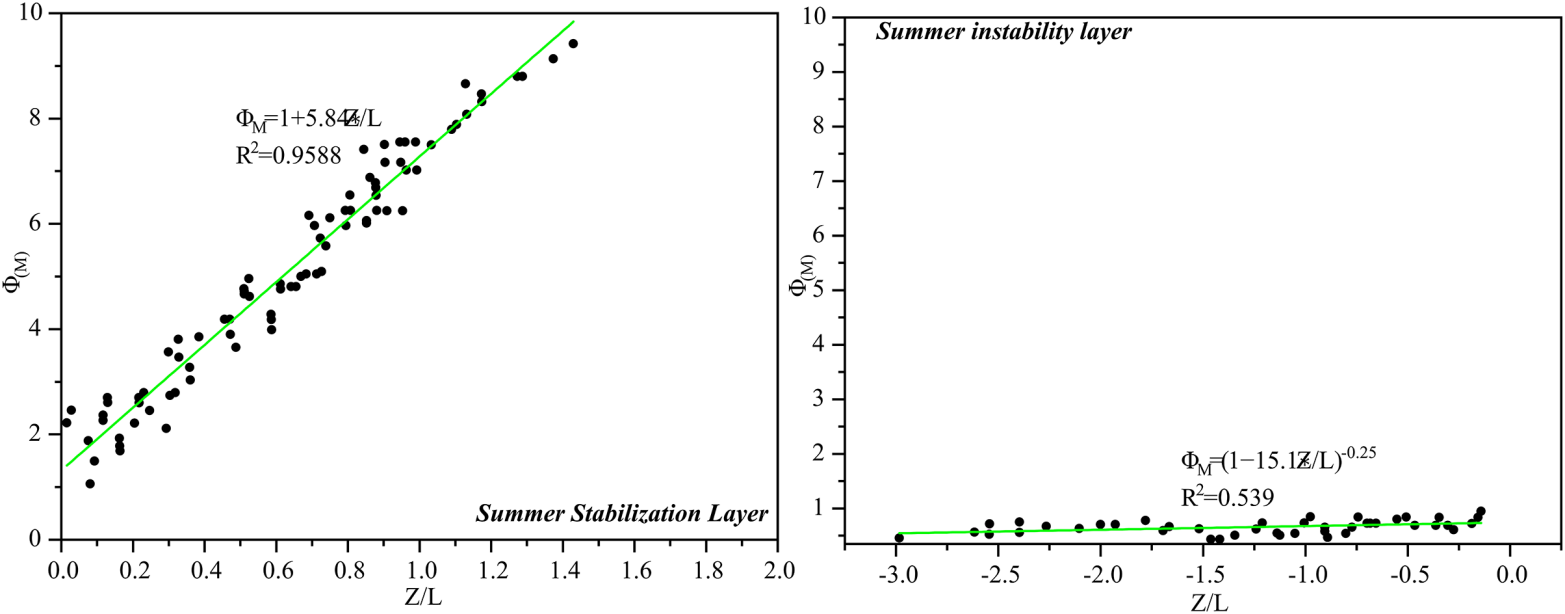
The relationship between surface strata ${\phi _m}$ and ${z \over L}$ at Tazhong tower in summer.

Figure 7 shows the distribution of ${\varphi_m}$ with stability under stable and unstable summer conditions in the middle of the tower. The parameters ${\gamma _m}$ and ${\beta _m}$ for the surface strata in the middle of the tower in summer were fitted by Eq. (7) and were 5.84 and 15.1.

**Figure 8:**
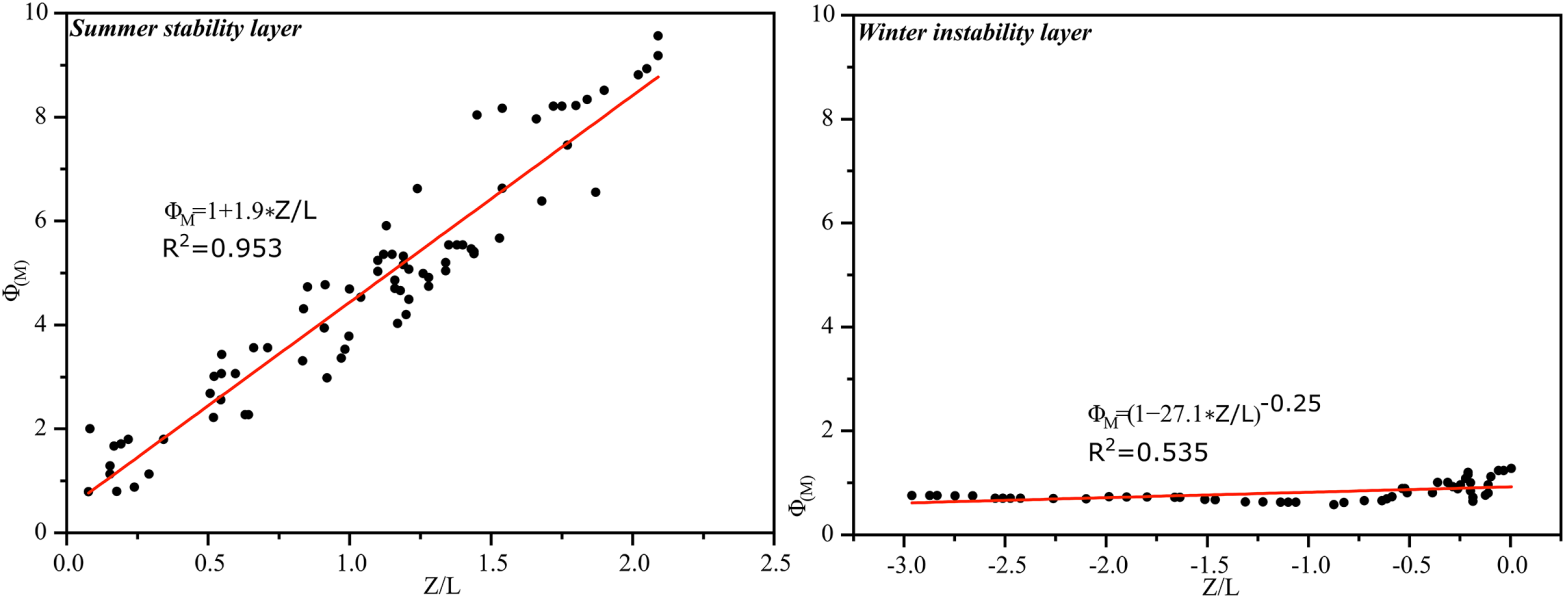
The relationship between surface strata ${\phi _m}$ and ${z \over L}$ at Tazhong tower in winter.

Using the same treatment as in summer, the relationship between near-surface strata ${\varphi_m}$ and ${z \over L}$ in the hinterland of Tazhong Desert was also fitted in winter, and the measured parameters ${\gamma _m}$ and ${\beta _m}$ were obtained. Figure 8 shows the relationship. The surface layer parameters ${\gamma _m}$ and ${\beta _m}$ in the middle of the tower in winter were obtained by fitting and had values of 1.9 and 27.1, respectively.

## Discussion

Vegetation in desert areas can improve the local microclimate. Desert plants regulate the microclimate in different ways, such as reducing surface wind speed, cooling, and humidification. Many restored green spaces have been constructed in the Tarim Basin oilfield. Compared with Xiaotang, wind speed, temperature, and other meteorological indicators were different in the Tarim Basin, mainly due to the inverse temperature and humidity conditions in winter. The main causes of this phenomenon were the high daytime temperature and low relative humidity of the desert. The water vapor from diffusion at night migrated to the oasis-desert transition zone and increased its relative humidity; thus, forming a circulation mechanism. Vegetation and shelter forest reduced the solar radiation reaching the surface and the emission of long-wave radiation from the ground. The airflow into the canopy was blocked by vegetation, weakening the intensity of heat exchange, resulting in temperature differences between shelter forests, transition zones, and desert areas. Protective forests and vegetation reduced diurnal temperature variations, keeping temperatures relatively stable. In the hinterland of the Taklimakan Desert, due to the continuous expansion of restored green spaces and the enhancement of transpiration from restored vegetation, the relative humidity, temperature, surface temperature, and ground temperature of the near-surface layer of restored green spaces inevitably decreased; thus, changing the hydrothermal conditions of the desert. Due to the influence of vegetation, the increase in ground moisture reduced the surface reflectance, increased the absorption of surface radiation, increased the radiation balance value, and slowed down the temperature change. In the growing season, restored green spaces reduced the surface wind speed, maintain water and soil conditions, and reduced the extent of erosion caused by wind-sand disaster weather.

## Conclusion

By analyzing the diurnal variations of the mean wind speed and temperature on sunny days and the characteristics of different wind speed profiles in the middle of observation towers at Tazhong and Xiaotang in summer, autumn, winter, and spring of 2014 and 2015, the following conclusions were reached.

In the four seasons, the diurnal variation of average wind speed on sunny days at different heights in the surface layer of Tazhong and Xiaotang had two characteristics. First, there were two peaks and two low values in the diurnal variation of wind speed. The two peaks occurred at night and in the daytime, and the two low values occurred in the morning and evening, respectively. However, the wind speed varied and the time of occurrence of maximum values differed among the seasons. Second, there were two distinct forms of change in the lower and upper layers. In the lower layer, the daytime wind speed was greater than in the night, in the upper layer the nighttime wind speed was greater than during the daytime, and the middle layer was stable, but there were differences in each season the diurnal variation of wind speed in autumn and spring was larger in Tazhong than in Xiaotang, whereas the variation was similar in summer and winter.

When a semi-empirical theory was applied, under neutral stratification there was a high correlation coefficient when a logarithmic law was used for the fitting, and the timing of the occurrence of a neutral atmosphere was consistent with the transition time of the temperature profile. Under non-neutral stratification, the wind speed profile deviated from the logarithmic law, and the fitting was better for an exponential law. For the M–O wind speed profile model, when the atmosphere was close to a neutral stratification and when the general function ${\varphi_M}\left( {{{\rm z} \over L}} \right) \to 1$ approached 1, the near-neutral stratification was fitted by a “logarithmic + linear” profile, and the results obtained were ideal. The ${\gamma _m}$, and ${\beta _m}$ values under stable and unstable stratification in the hinterland of the Tazhong Desert were 5.84 and 15.1 in summer and 1.9 and 27.1 in winter, respectively.

## Supplemental Information

10.7717/peerj.13001/supp-1 Supplemental Information 1 Raw data.

## Additional Information and Declarations

### Data Availability

The following information was supplied regarding data availability: The data is available at figshare: Yongde, Kang (2022): Data.xls. figshare. Dataset. https://doi.org/10.6084/m9.figshare.19253594.v1.
